# Epigenomic characterization of a p53-regulated 3p22.2 tumor suppressor that inhibits STAT3 phosphorylation via protein docking and is frequently methylated in esophageal and other carcinomas: Erratum

**DOI:** 10.7150/thno.70351

**Published:** 2022-01-21

**Authors:** Lili Li, Juan Xu, Guohua Qiu, Jianming Ying, Zhenfang Du, Tingxiu Xiang, Kai Yau Wong, Gopesh Srivastava, Xiao-Feng Zhu, Tony S Mok, Anthony TC Chan, Francis KL Chan, Richard F Ambinder, Qian Tao

**Affiliations:** 1 Cancer Epigenetics Laboratory, Department of Clinical Oncology, State Key Laboratory of Oncology in South China, Sir YK Pao Center for Cancer and Li Ka Shing Institute of Health Sciences, The Chinese University of Hong Kong, Hong Kong;; 2 Shenzhen Second People's Hospital, the First Affiliated Hospital of Shenzhen University, Shenzhen, China;; 3 Johns Hopkins Singapore, Singapore;; 4 Department of Pathology, National Cancer Center/Cancer Hospital, Chinese Academy of Medical Sciences & Peking Union Medical College, Beijing, China;; 5 Chongqing Key Laboratory of Molecular Oncology and Epigenetics, The First Affiliated Hospital of Chongqing Medical University, Chongqing, China;; 6 Department of Pathology, Queen Mary Hospital, The University of Hong Kong;; 7 State Key Laboratory of Oncology in South China, Sun Yat-sen University Cancer Center, Guangzhou, China;; 8 Institute of Digestive Disease and State Key Laboratory of Digestive Diseases, Department of Medicine and Therapeutics, The Chinese University of Hong Kong;; 9 Sidney Kimmel Comprehensive Cancer Center, Johns Hopkins School of Medicine, Baltimore

In the original publication, errors were found in Fig 3 and Suppl 8A. During the assembling of figures, in Fig 3A, the colony formation assay photos of KYSE150 were misused with re-scanned KYSE510 photos; in Fig 3B and Suppl 8A, the Western blot photos of H1299 were swapped with that of HONE1 for each other. The correct figures are shown below. The authors confirm that these corrections do not change the result interpretation or conclusions of the article. The authors are deeply sorry and sincerely apologize for any inconvenience or misunderstanding that may have caused.

## Figures and Tables

**Figure 1:**
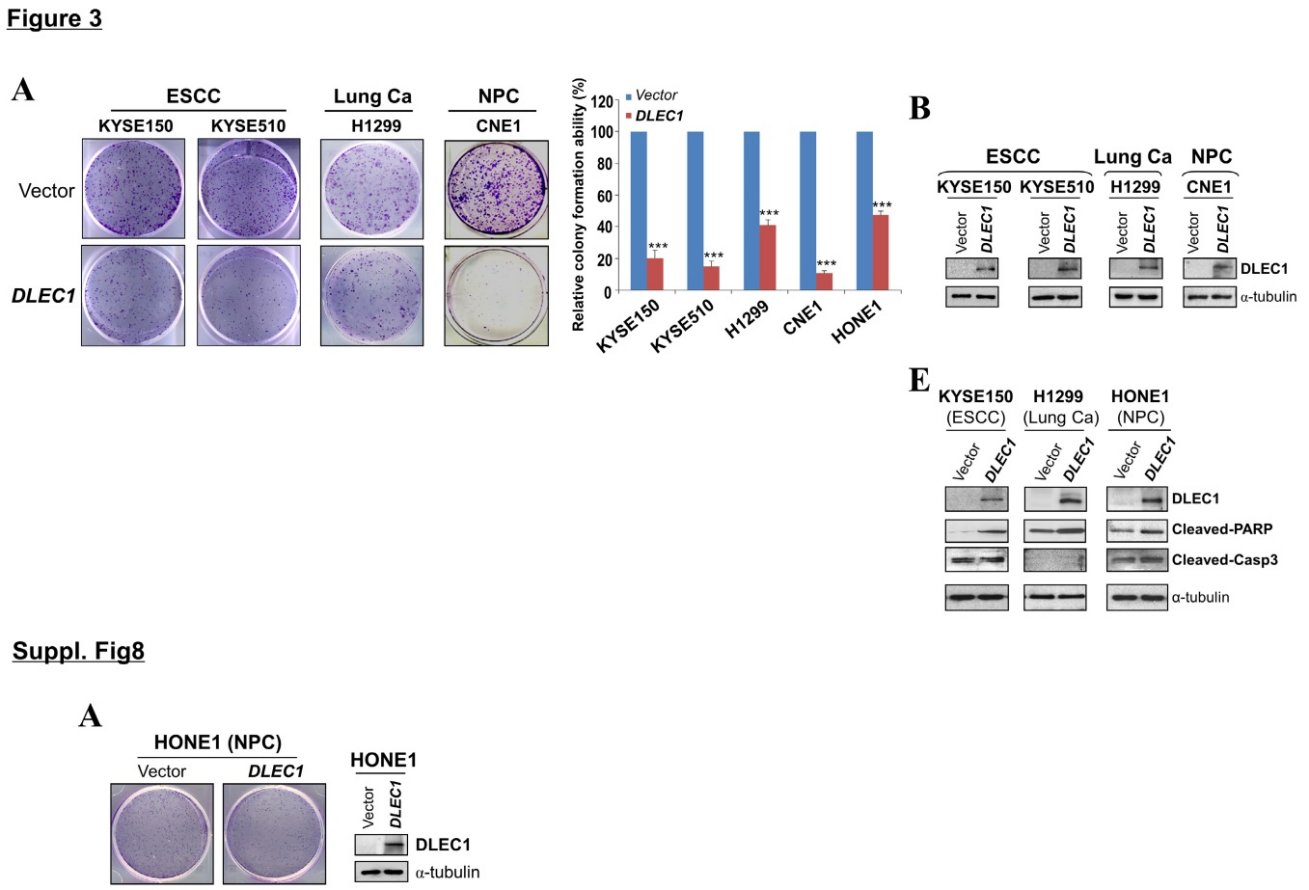
Corrected Figure 3 and Suppl. Figure 8.

